# MR Vascular Fingerprinting with Hybrid Gradient–Spin Echo Dynamic Susceptibility Contrast MRI for Characterization of Microvasculature in Gliomas

**DOI:** 10.3390/cancers15072180

**Published:** 2023-04-06

**Authors:** Krishnapriya Venugopal, Fatemeh Arzanforoosh, Daniëlle van Dorth, Marion Smits, Matthias J. P. van Osch, Juan A. Hernandez-Tamames, Esther A. H. Warnert, Dirk H. J. Poot

**Affiliations:** 1 Department of Radiology and Nuclear Medicine, Erasmus MC, University Medical Center Rotterdam, 3015 GD Rotterdam, The Netherlands; 2 Erasmus MC Cancer Institute, Erasmus MC, 3015 GD Rotterdam, The Netherlands; 3 C. J. Gorter MRI Center, Department of Radiology, Leiden University Medical Center, 2333 ZA Leiden, The Netherlands; 4 Department of Medical Imaging, Faculty of Applied Physics, Delft University of Technology, 2628 CD Delft, The Netherlands

**Keywords:** MR vascular fingerprinting, dynamic susceptibility contrast imaging, glioma, vascular biomarkers, vessel radius, cerebral blood volume, vessel size imaging

## Abstract

**Simple Summary:**

Primary brain tumors, most commonly gliomas, are devastating diseases which in adults are generally fatal. Vascularization is an important aspect of the biological behavior of gliomas, and determining it is valuable for the optimal timing of treatment. Magnetic resonance imaging (MRI) is an excellent non-invasive diagnostic technique for tissue characterization. In this study, we propose an advanced MRI technique, MR vascular fingerprinting based on the dynamic passage of a contrast agent, to gather quantitative information on the major vascular biomarkers of gliomas within an acceptable scan time. This technique was evaluated in six patients with gliomas, obtaining the vascular parameters that deliver information on the vascularity of the tumor. The vessel parameters quantified using the proposed technique were also compared to those quantified using a conventional vessel size imaging technique. This study will significantly contribute to further advances in functional imaging for gliomas.

**Abstract:**

Characterization of tumor microvasculature is important in tumor assessment and studying treatment response. This is possible by acquiring vascular biomarkers with magnetic resonance imaging (MRI) based on dynamic susceptibility contrast (DSC). We propose magnetic resonance vascular fingerprinting (MRVF) for hybrid echo planar imaging (HEPI) acquired during the first passage of the contrast agent (CA). The proposed approach was evaluated in patients with gliomas, and we simultaneously estimated vessel radius and relative cerebral blood volume. These parameters were also compared to the respective values estimated using the previously introduced vessel size imaging (VSI) technique. The results of both methods were found to be consistent. MRVF was also found to be robust to noise in the estimation of the parameters. DSC-HEPI-based MRVF provides characterization of microvasculature in gliomas with a short acquisition time and can be further improved in several ways to increase our understanding of tumor physiology.

## 1. Introduction

Magnetic resonance imaging (MRI) using dynamic susceptibility contrast (DSC) is a well-established, clinically applied method used to assess the vasculature of primary brain tumors such as gliomas [1,2,3]. Previous studies demonstrated that characterizing tumor microvasculature can provide important information for prognosis in glioma cases [4]. Vascular parameters derived from DSC-MRI are utilized as image-based biomarkers for treatment management of patients with gliomas. Relative cerebral blood volume (rCBV) and vessel radius (R), which are the most widely used parameters derived from DSC-MRI for predicting the grade of tumor and survival of the patient, are of particular interest [5,6]. rCBV delivers information on the vascularity of the tumor [7]. Law et al. demonstrated how rCBV is related to the various attributes of microvasculature and the study of tumor tissues’ biological behavior [8]. Vessel size estimation is emerging as a method for characterizing angiogenesis beyond blood volume fractions in tumor models [9].

The vessel architecture of tissue can be assessed by combining spin echo (SE) and gradient echo (GRE) image acquisitions. T2- and T2*-weighted images acquired during DSC imaging can be used to examine microvasculature and the combination of microvasculature and larger vessels by exploiting the differences in transverse relaxation rates R2 and R2*, respectively, during the passage of contrast agent (CA) through the vasculature [10,11,12]. Hybrid-EPI (HEPI) is a fast acquisition technique for simultaneous GRE and SE acquisitions, which allows for imaging vasculature from a single bolus injection [13].

An analytical model for estimating vessel size from GRE and SE signals was established by Kiselev et al. [14,15]. This model allows for quantitative vessel size estimations and requires information on the local diffusion coefficient and rCBV. While the results have been encouraging, recent studies have shown that the existing analytical models can be inaccurate in describing the MR signal due to the difficulty in characterizing the complex influence the process of water diffusion has in the presence of noise [16]. To overcome such difficulties of analytical modeling, Christen et al. introduced an approach using numerical simulations and dictionary matching [17]. This technique is called MR vascular fingerprinting (MRVF) and is combined with an analysis of SE signal evolutions during the pre- and post-contrast phases and free induction decay to retrieve quantitative information about the microvasculature. Their numerical simulation tool considers a virtual voxel containing blood vessels [18]. It takes an apparent diffusion coefficient (ADC) as the input and models the water diffusion effects through the magnetic field disturbances caused by the CA and calculates the MR signal evolution from this voxel. MRI fingerprinting approaches based on dictionary matching are also known to be robust to measurement noise [19].

In the current work, we propose an MRVF approach to disentangling vascular biomarkers, notably, the rCBV and vessel radius (R), using the HEPI sequence during bolus injection in patients with gliomas. We tailored the MRVF technique proposed by Christen et al. to the HEPI sequence [17]. This allows vascular information to be acquired from the bolus passage, rather than only using the static pre- and post-contrast injection phases. A dictionary of HEPI signal evolutions was generated in which rCBV, vessel radius and permeability were varied in the simulated vasculature. To retrieve vascular parameter maps, the best match between the acquired HEPI images and the dictionary was obtained for each voxel. We also quantified the same data using the analytical vessel size imaging (VSI) [14] post-processing technique to compare the performance of our fingerprinting approach to this conventional approach in quantifying clinically acquired data of patients diagnosed with glioma.

## 2. Materials and Methods

### 2.1. Data Acquisition

A dataset containing HEPI-DSC MRI in six patients (two women, four men; mean age 38 years) with a confirmed diagnosis of glioma was used retrospectively in this study. The study was approved by the Institutional Medical Ethical Committee and all patients provided informed consent. Data were collected, prior to surgery, at the Erasmus MC (Rotterdam, The Netherlands) with a 3T MRI scanner (MR750, GE, Milwaukee, WI, USA) using a 32-channel head coil. A bolus of 7.5 mL of gadolinium-based contrast agent (GBCA; Gadovist, Bayer, Leverkussen, DE) was administered to the patients just before the acquisition with a 2D HEPI sequence (Repetition time, TR: 1500 ms, Echo times, TE_GRE_: 18.6 ms and TE_SE_: 69 ms, voxel size: 1.9 × 1.9 × 3 mm^3^ and 1 mm gap between slices, field of view (FOV): 24 × 24 × 6 cm, number of slices: 15 and number of scans: 120).

A preload injection of the same dose was given 5 min prior to the HEPI scan. High-resolution structural images of 3D T1-weighted MPRAGE sequences (TR: 6.1 ms, TE: 2.1 ms, Inversion time, TI: 450 ms, voxel size: 1.0 × 1.0 × 0.5 mm^3^, FOV: 25.6 × 25.6 × 16.6 cm, total scan time: 4 min and 35 s), 2D T2-weighted PROPELLER sequences (TR: 10,000 ms, TE: 107 ms, voxel size: 0.5 × 0.5 × 3.3 mm^3^, FOV: 22 × 22 × 14.8 cm, number of slices: 45, slice thickness: 3 mm, with 0.3 mm gap between slices, total scan time: 4 min and 35 s) and 3D T2-weighted fluid-attenuated inversion recovery (FLAIR; TR: 6000 ms, TE: 112 ms, TI: 1890 ms, voxel size: 0.8 × 0.5 × 0.5 mm^3^, FOV: 16.6× 25.6 × 25.6 cm, total scan time: 4 min and 35 s) were also acquired as part of routine clinical imaging. T1-weighted scans were collected both before GBCA injection (pre-contrast T1-weighted) and after injection of the preload bolus but prior to the HEPI scan (post-contrast T1-weighted) to identify enhanced tumor tissue. A diffusion-weighted scan, used for estimation of the ADC as required for vessel size measurements, was also included in the protocol with the following acquisition parameters, all of which were used for the calculation of ADC: TR: 5000 ms, TE: 63 ms, voxel size of 1.0 × 1.0 × 3.0 mm^3^; FOV: 25.6 × 25.6 × 14.4 cm, number of slices: 48, slice thickness: 3 mm, with no gap between slices and 3 isotropic diffusion weighting gradients of 0,10,1000 s/mm^2^ strength (b values). For tumor segmentation, the structural images of T1-weighted pre-contrast, T2-weighted and FLAIR were registered to T1-weighted post-contrast using the Elastix toolbox (version 2.5) [20]. Based on these structural images, the tumor masks for gliomas were delineated using HD-GLIO [21,22]. Normal-appearing white and gray matter masks (NWMs, NGMs) were generated from the pre-contrast T1-weighted high-resolution structural scan using FAST (FSL v. 6.01.1, Oxford, UK).

### 2.2. Simulation of MR Signal

**Figure 1:**
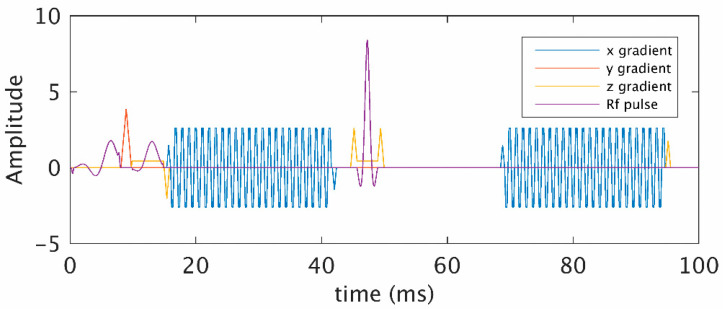
Hybrid echo planar imaging (HEPI) pulse sequence (waveforms and timings) obtained from the scanner. Gradient echo (GRE) was acquired after the first excitation pulse at echo time, at 20 ms, and spin echo (SE) was acquired with at echo time, 70 ms after the refocusing pulse.

A 2D simulation tool (DCESim) was employed in this study, which used a numerical approach to simulate the MR signal based on the Bloch equations, modeling the magnetic field perturbations, diffusion effects of water and Cas for an input voxel that contains blood vessels [18]. We recorded the acquisition details of the HEPI sequence (as shown in the sequence plotter in Figure 1) from the same scanner with which the patients were scanned. The pulse sequence was exported from the scanner and imported into the simulation tool, considering all the timing features (that including the excitation time, repetition time, echo times of GRE and SE), actual waveforms and amplitudes of radiofrequency pulses and gradients. The complex magnetization changes were calculated per time step (Δt = 1 ms) and integrated over the simulation volume to obtain the simulated GRE and SE HEPI signal, as described in the original description of the model [18].

We simulated 5 vessels of radius, R, that occupied a fraction of the simulation volume (128 × 128 points) given by the rCBV. The permeability (k) controls the exchange of the CA between the vessels and its peripheral space. In addition to the default values of the input parameters specified in the simulation tool, certain parameters were assigned values as follows: static magnetic field (B_0_), 3T, water diffusion coefficient, 1000 μm^2^ s^−1^, hematocrit value, 40% and oxygen saturation, 60%. The arterial input function (AIF) for our experiments was modeled as a function defined by Parker et al. [23]. We simulated 600 s of spin evolution, which included a baseline of 20 s and then a preload of the CA followed by a 280 s delay before the main bolus was virtually injected.

### 2.3. Dictionary Generation and Matching

**Figure 2:**
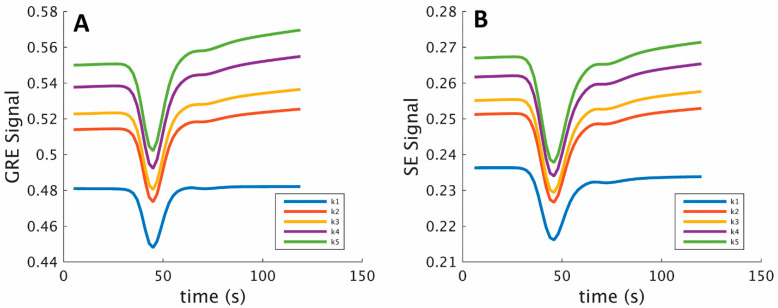
GRE (**A**) and SE (**B**) signals for 5 atoms from the simulated dictionary for different permeabilities, k (k1 = 0, k2 = 0.3 × 10^−3^ s^−1^, k3 = 0.4 × 10^−3^ s^−1^, k4 = 0.6 × 10^−3^ s^−1^, k5 = 0.8 × 10^−3^ s^−1^) and a fixed vessel radius (R = 87 µm) and relative cerebral blood volume (rCBV = 4.6%) during the bolus injection, after the preload.

The dictionary of HEPI signal evolutions was generated for a grid with 50 logarithmically spaced values of R ([5, 150] μm), 40 logarithmically spaced values of rCBV ([0.5, 10] %) and 10 values of k (9 logarithmically spaced values between [2.5, 6] × 10^−3^ s^−1^ and 0). Each of the 20,000 atoms in the dictionary provided a vascular fingerprint consisting of both the GRE and SE signals for a particular combination of vessel parameters (k, R, rCBV). Figure 2 shows a subset of the HEPI dictionary with its GRE and SE signals for specific k, R and rCBV values. The HEPI data acquired from the patients were subsequently matched with the dictionary. To synchronize the to-be-matched dictionary with the time of CA injection as present in the data, a single delay was estimated. Specifically, a delay that minimized the mean square deviation between the matched and acquired HEPI time series in the tumor region of interest (ROI) was selected. As described by Valenberg et al., the matching was carried out using a separate scaling factor for the GRE and SE parts to compensate for baseline signal differences between the dictionary and in vivo signals [24]. The vessel parameters corresponding to the match from the dictionary to the data were returned.

### 2.4. Comparison with Vessel Size Imaging

For the VSI technique, rCBV maps were calculated using the GRE data from HEPI by estimating the trapezoidal integration of the relaxivity–time curve [2,25]. Estimates of the mean vessel radius for each voxel were obtained by

(1) $$Vessel\;Size=0.86\times {\left(rCBV\times ADC\right)}^{1/2}\times ((∆{R2}^{*})/\left({∆R2)}^{3/2}\right)$$

where ADC is the water diffusion coefficient (mm^2^ s^−1^), rCBV is the relative cerebral blood volume scaled to the median value in normal-appearing matter of 3.2%, and ΔR2* and ΔR2 are transverse relaxation rates acquired from GRE-DSC and SE-DSC, respectively [15]. A leakage correction algorithm was implemented in the measurements of rCBV, ΔR2* and ΔR2 [26,27].

Vessel radius and rCBV parameters obtained via dictionary matching were compared with those estimated with the VSI technique data by assessing the Structural Similarity Index Measure (SSIM) between the parameter maps. The average R and rCBV values estimated using both techniques in the whole brain and tumor regions were also quantitatively compared through a Bland–Altman analysis.

### 2.5. Noise Analysis

Two experiments analyzing noise performance were performed: one to examine the reliability of the dictionary matching process and the other to compare the techniques (MRVF and VSI) in terms of robustness to noise. First, a Monte Carlo experiment with 100 iterations was performed by applying the fingerprinting approach to a synthetic image built from the dictionary with noise added to it (with an SNR of 38dB for GRE and 32dB for SE). These synthesized images were subsequently matched to the dictionary to retrieve the vessel parameters. From the 100 realizations, the mean and standard deviation (SD) of the R and rCBV were calculated.

Secondly, to compare both techniques, independent Gaussian random noise was added to the 6 patient datasets (with an average signal-to-noise ratio, SNR, of 27dB for GRE and 22dB for SE), and the noisy data were matched with the dictionary in MRVF to evaluate their influence on the obtained vessel parameters. This was subsequently repeated but using VSI, as described above. To examine the robustness to noise, the absolute differences between the parameter maps obtained from the raw and noisy data were determined, and the root mean square deviation (RMSD) was evaluated for each parameter and dataset for both techniques.

## 3. Results

### 3.1. Parametric Maps

The vessel parameters, k, R and rCBV, were retrieved for each dataset via dictionary matching. Table 1 shows the patient information along with the diagnosis, tumor grade and molecular profile (based on the 2021 WHO classification of tumors) as well as the mean and SD of the vessel parameter values obtained in the tumor region for the each of the subjects. It can be observed that comparatively higher k values were obtained in the enhancing tumor voxels among all the six patients.

**Figure 3:**
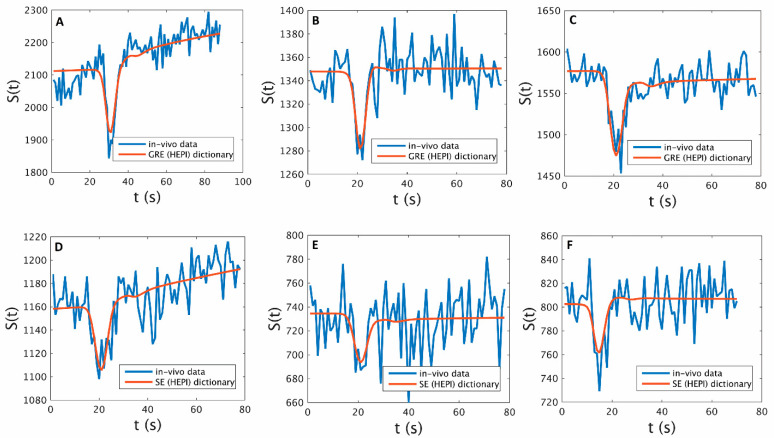
Signals from a tumor voxel in patients 1, 2 and 3 from GRE ((**A**,**B**,**C**), respectively) and SE ((**D**,**E**,**F**), respectively) images (blue) and their best match according to the respective GRE and SE of the HEPI dictionary (red).

**Figure 4:**
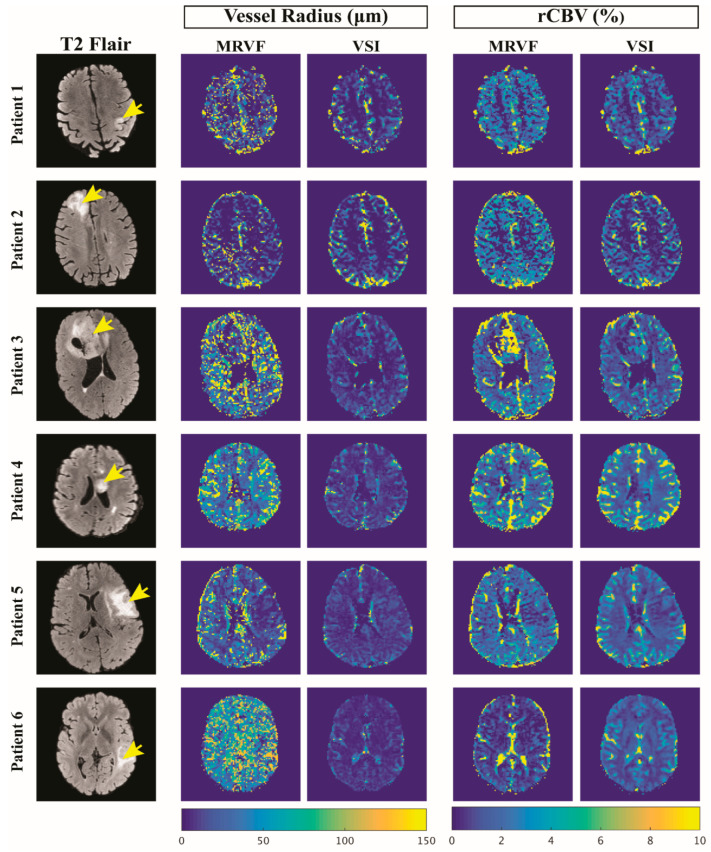
T2 FLAIR images of 6 patient datasets of slices showing the glioma (marked by the yellow arrows) and the corresponding vessel radius (in μm) and rCBV (in %) maps obtained using the MRVF and the VSI methods.

**Figure 5:**
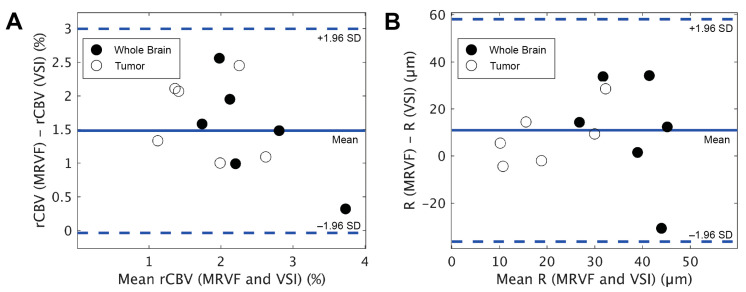
Bland–Altman plots resulting from the comparison of mean rCBV (**A**) and R (**B**) obtained in the whole brain and tumor regions from the MRVF and VSI techniques for the 6 patients.

The GRE and SE time series and their dictionary match for tumor voxels in three example subjects are shown in Figure 3, and the graphs show the best match obtained from the dictionary for the tumor voxels. The rCBV and vessel radius parameter maps obtained with MRVF and VSI for all subjects are shown in Figure 4. The rCBV maps obtained via the MRVF technique clearly distinguish between the gray matter and white matter. Both techniques showed comparable patterns, although there were a few qualitative differences especially in patient 3, whose tumor had a high rCBV in the MRVF technique but not in VSI. The SSIM between MRVF and VSI in the R and rCBV for all subjects is presented in Table 2, and it can be observed that both techniques led to similar results as evidenced by the moderate-to-high SSIM values. The results of the Bland–Altman agreement analysis between the two techniques for mean parameter values in the whole brain and tumor regions for the six patients are shown in Figure 5. The analysis revealed a mean difference of 1.48% with the limits of agreement ranging from −0.03% to 3% for rCBV (*p* < 0.05 obtained with a paired t test performed between the mean rCBV between MRVF and VSI). For R, a mean difference of 10.9 µm with the limits of agreement ranging from −36.24 µm to 58.05 µm (*p* = 0.91 obtained with paired t test between the average R between the two techniques) was observed. This analysis showed a significant difference in rCBV between the techniques, while the difference was not significant for R with the MRVF and VSI.

### 3.2. Noise Analysis

**Figure 6:**
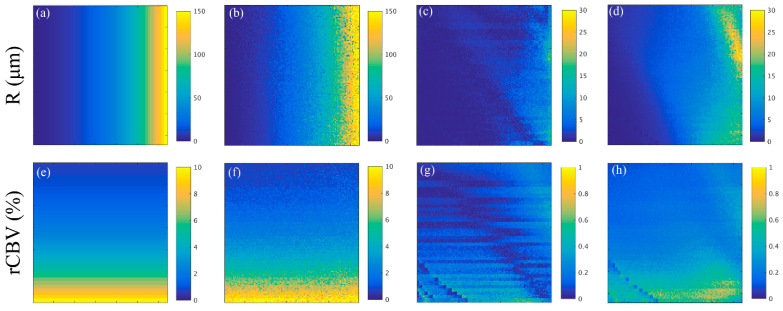
(**a**) and (**e**) are the vessel radius, R (in μm) and rCBV (in %) maps of the synthetic image from the dictionary, respectively. R (in μm) and rCBV (in %) estimated from matching one of the noisy datum to the original dictionary are shown in (**b**) and (**f**), respectively. (**c**) shows the difference in R (in μm) between the values of the ground truth and the mean of the R estimates, and (**d**) is the standard deviation (in μm) of the R maps estimated from the 100 noise realizations. (**g**) corresponds to the difference between the rCBV (in %) values of the ground truth and the mean of the rCBV, and (**h**) gives the standard deviation of the rCBV (in %) obtained from the 100 noise realizations.

The MRVF approach was applied to the noise-added atoms from the dictionary to test the accuracy of the proposed technique. Figure 6a,e show the ground truth R and rCBV maps of the dictionary with which the test images were synthesized. Figure 6b,f show one realization of the R and rCBV parameters. Figure 6c,g show the difference in the ground truth of this realization. The average value of the difference between the rCBV values of the ground truth and mean rCBV values implies a bias of 0.079%, which is 3.3 μm for the vessel radius. The averages for the SD maps of rCBV (Figure 6h) and vessel radius (Figure 6d) were 0.3% and 10.4 μm, respectively. These low average values indicate that the parameters were estimated accurately even in the presence of random noise using the proposed approach.

**Figure 7:**
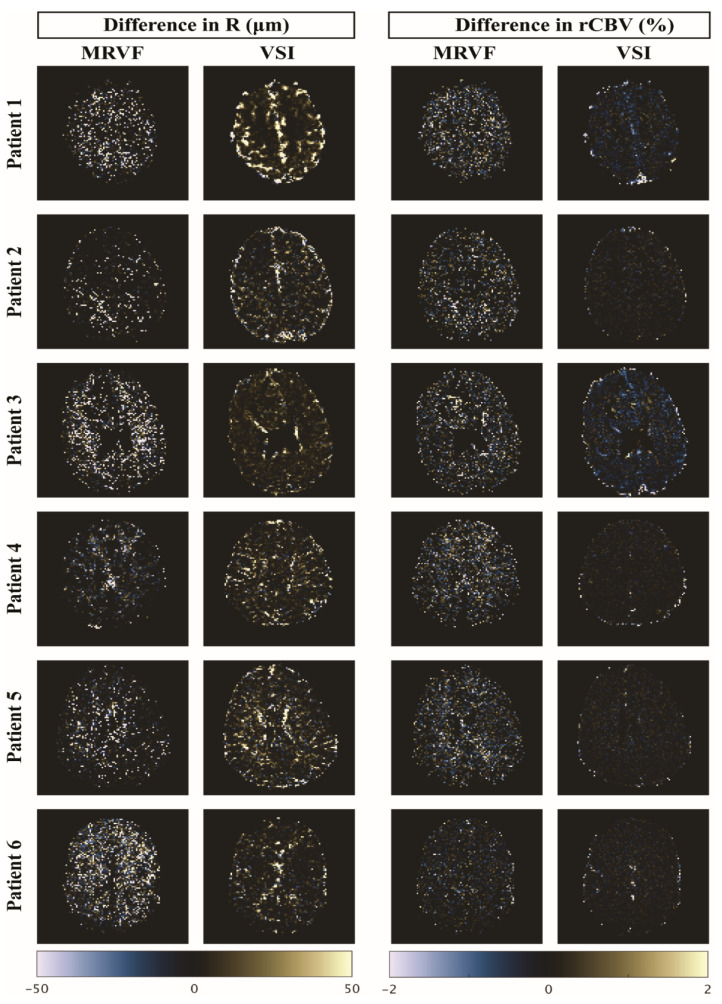
Difference maps of R (in µm) and rCBV (in %) estimated from raw and noisy data using MRVF and VSI for the 6 patient datasets.

For the comparison of robustness to noise between the techniques, Figure 7 shows the difference in R and rCBV maps estimated from the raw and noisy in vivo data for both techniques for all six datasets. Mean values of the parameter maps before adding noise are shown in Table 3 for both techniques, in the whole brain and tumor regions. Root mean square deviations (RMSDs) calculated between each of the parameter maps obtained before and after adding noise for the whole brain and tumor regions are shown in Table 4. The ratio of the RMSD to the average of R was found to be lower in MRVF in the whole brain (0.64) and the tumor (0.94) in comparison to that in the VSI (1.97 in the whole brain and 0.99 in the tumor). The ratio of RMSD to the respective mean in rCBV was also lower in MRVF, with the ratios being 0.27 in the whole brain and 0.29 in the tumor compared to the VSI (where the ratios were 0.55 and 0.32 in the whole brain and the tumor, respectively). This analysis implies that the relative error of rCBV is lower than that of R in the presence of noise, while MRVF is relatively more robust to noise than the VSI in the estimation of both parameters.

## 4. Discussion

In this study, we propose a DSC-HEPI-based vascular fingerprinting approach to quantitatively characterizing microvasculature in gliomas. We successfully imported the HEPI sequence as run on the scanner into a simulation tool, included a preload bolus, built a dictionary of simulated DSC-HEPI signals and matched these to in vivo data to retrieve vascular parameters in six patients with gliomas. The major findings of this study are as follows: (1) it quantified the major vascular biomarkers, rCBV and vessel radius in tumors and normal tissues, even when tumorous tissue had a leaky blood–brain barrier; the obtained values agreed well with those acquired via the conventional VSI technique, while being more resilient to noise and CA leakage; (2) different to the previously published MRVF method [17], our approach exploits the dynamic phase during and after CA injection that is monitored by the fast HEPI technique, thereby providing additional microvasculature information within a short scan time.

The MRVF approach is based on simulating the MRI signal within the microvasculature during the passage of the CA. With these simulations, a dictionary is created that is employed in a matching step to translate measurements into vascular parameter maps. This simulation-based approach has a major advantage in that it allows more physiological and MRI signal formation effects to be taken into consideration than allowed by analytical models. For example, when the CA leaks into the extravascular space, it leads to T1 shortening and thus signal enhancement as well as reduced susceptibility. Furthermore, the contrast-induced signal intensity drops. In our simulations, these effects induced by the extravasation of the CA were included and hence corrected for in the vascular parameter maps, while an additional leakage correction technique is required for VSI [28].

Although both techniques (MRVF and VSI) yielded similar rCBV maps for the gray and white matter in all patients (Table 2), a significant quantitative difference was obtained in one patient (Figure 4) with an enhancing glioma. Although our VSI implementation did include a leakage correction, this was previously proven to be sub-optimal in high-grade tumors with high vascular permeability [29,30,31]. The dictionary in MRVF did, however, include permeability as an input parameter for the simulations, and this might explain the observed differences in rCBV in the tumor region. For this reason, we favor MRVF, as CA leakage is explicitly included in the simulation. The use of a preload dose minimizes leakage effects, although it has been shown previously that preloading could lead to an underestimation of rCBV [32]. The fact that patient 3 had a different glioma type and molecular profile compared to the other two patients with enhancing tumors (Table 1) could also be a possible factor for this significant difference in parameter maps between the two techniques. Since the current study was limited to only three patients with an enhancing tumor, the factors that cause differences in rCBV values warrant further investigation by applying the technique to more datasets and preferably with a comparison to a gold-standard measurement, which is unfortunately difficult to obtain.

The vessel radius maps obtained for each dataset from MRVF were very similar to the estimations obtained using VSI, and both techniques clearly differentiated the different regions of the brain. According to Kellner et al. [15], VSI has a limitation of underestimation of larger vessel sizes, and this difference in sensitivity to larger vessels could explain the low-intensity vessel radius maps obtained from VSI compared to those from MRVF. The vessel radius maps obtained from MRVF are noisier than those from VSI. This could be explained by the fact that the simulated signals showed little variation for vessel radii above 60 μm, i.e., in the regime where SE is less sensitive [3,11]. This implies that the same insensitivity to larger vessels results in a different reflection in the parameter maps for both approaches. Less dense sampling in this part of the dictionary might therefore be possible without loss of accuracy and result in less noisy maps, although the observer should be aware that the homogenous nature of the image is still due to poor sensitivity. A further improvement to our dictionary matching procedure would be to allow interpolation between atoms [24]. This could also be employed to reduce dictionary generation time via less dense sampling of the parameter space. Finally, the MRVF technique was also found to be accurate in estimating the vessel parameters in the presence of noise (Figure 6 and Figure 7), as evidenced by the relatively low RMSD values (Table 4).

Parameter mapping using DSC-HEPI-based MRVF has an advantage in that it acquires data during the dynamic phase of the bolus passage of the CA, which potentially yields more information on microvasculature than traditional MRVF [17]. Traditional MRVF is a steady-state approach that acquires signals during pre- and post-contrast phases and is especially employed in combination with a CA based on iron oxide [17]. Iron-based CAs improve the SNR but are also known to have several disadvantages [33], whereas our work used the more commonly employed gadolinium-based CA. By scanning during the dynamic bolus passage, the concentration of the contrast agent, and thus the magnitude of magnetic susceptibility effects, will cover a wide range, which is different from the previous MRVF procedure [17], in which only a single concentration is measured besides the pre-contrast measurements. To compensate for this lack of variation in concentrations in the sequence, traditional MRVF measures a wider range of echo times, whereas in our approach, only a single GE and SE are recorded. Since conventional DSC-MRI forms part of standard clinical tumor protocols and our HEPI technique is essentially a DSC sequence, both traditional DSC analysis can be performed as well as our MRVF approach. Hence, our approach does not require additional acquisition time. Of course, MRVF processing will provide additional maps, such as the mean vessel radius [3,4,34]. However, a major constraint of DSC-based MRVF is that a temporal resolution of 1.5–2 s is needed, which limits the spatial resolution as well as the coverage that can be achieved.

There are some limitations in our current implementation. First, the proposed DSC-MRVF approach assumes a single shape for the AIF, irrespective of the region of the brain, and in its current form, it cannot correct for differences in arrival time of the CA bolus for different tissues [35]. During the matching procedure, we chose the best delay such that the RMSD was minimum for the tumor ROI. However, this only yielded a single delay for the whole scan. The dictionary matching could therefore be further improved by performing a regional or voxel-wise estimation of the delay, but the SNR of our data was not sufficient to achieve a more local delay estimation. Another limitation is that the computation time required to create the four-dimensional dictionary in the current tool is quite long (3 CPU-core hours per atom), though the computational complexity can likely be reduced with improved algorithm design. Additionally, we chose to include the preload injection and delay time in the simulations, which could be replaced by a more efficient, albeit less realistic, approach. Another drawback is that the current simulations are 2D, whereas a 3D version would be more realistic and could, for example, provide advanced options such as choice of vessel shapes and orientation at the expense of increased computation time. Subsequently, in this study, only the magnitude of the signal evolution was analyzed, thus ignoring the phase evolution, which could provide valuable information on the magnetic susceptibility distribution during the CA passage [17,36]. However, it is not trivial to include such phase evolutions in the dictionary, because these will be dependent on more large-scale form factors than present in the current microstructural simulation model. The number of vascular parameters varied to simulate the signal was limited to three in our study, while more parameters could influence the MRI signal. The simulation and dictionary could be extended to include more vascular parameters such as oxygen saturation, different diffusion values, tortuosity, etc., which would improve the realism of the simulations and might assist us in understanding tumor hemodynamics. More parameters in the dictionary, on the other hand, could also lead to poorer estimations with high sensitivity to imaging artifacts and would require even more computation time. Further research could focus on the extension of the technique to measure oxygenation in gliomas using HEPI-based MRVF [37].

## 5. Conclusions

In summary, DSC-HEPI-based MRVF provides quantification of important vascular biomarkers of gliomas during the first passage of the bolus and thus without significant examination times, as compared to traditional MRVF [17]. The vessel parameters retrieved, vessel radius and rCBV, were comparable to those attained with VSI [14] while being more resilient to noise. Parameter estimation could be further extended by including more vascular or physiological biomarkers to further improve our understanding of tumor hemodynamics and vascular architecture.

## Figures and Tables

**Table 1:** Diagnosis of the patient datasets and the mean and standard deviation (SD) in the tumor region of the vessel parameters, k, R and rCBV, obtained from the best match with HEPI signals.

Subject	Age	Sex	Diagnosis (Grade)	Molecular Profile	Tumor Type	k (10^−3^ s^−1^)		R (µm)		rCBV (%)	
						Mean	SD	Mean	SD	Mean	SD
1	65	M	Glioblastoma (IV)	IDH wild type	Enhancing	1.5	2	12.88	14.84	2.49	1.55
2	54	M	Oligodendroglioma (III)	IDH mutant, 1p/19q co-deleted	Non-Enhancing	0.58	1.7	8.53	11.63	2.45	1.87
3	24	M	Oligodendroglioma (III)	IDH mutant, 1p/19q co-deleted	Enhancing	0.75	1.5	22.78	32.35	3.48	3.12
4	22	F	Oligodendroglioma (II)	IDH mutant, 1p/19q co-deleted	Non-Enhancing	0.41	1.2	34.64	29.36	3.17	1.38
5	37	M	Astrocytoma (III)	IDH mutant	Enhancing	0.5	1.4	17.83	20.75	2.42	0.85
6	28	F	Astrocytoma (II)	IDH mutant	Non-Enhancing	0.45	1.5	46.58	44.41	1.79	1.45

**Table 2:** Structural Similarity Index Measure (SSIM) between MR vascular fingerprinting (MRVF) and vessel size imaging (VSI) techniques in the estimation of vessel radius and rCBV maps for each subject.

Subject	SSIM	
	Vessel Radius	rCBV
1	0.78	0.78
2	0.72	0.77
3	0.77	0.77
4	0.88	0.81
5	0.79	0.79
6	0.89	0.79
Average	0.81	0.79
SD	0.07	0.02

**Table 3:** Mean values of the vessel radius (in μm) and rCBV (in %) maps, before adding noise, in the whole brain and tumor regions for MRVF and VSI techniques. The average and SD of the mean for each parameter and technique among the 6 subjects are also shown.

Subject	Mean of Vessel Radius (μm)				Mean of rCBV (%)			
	Whole Brain		Tumor		Whole Brain		Tumor	
	MRVF	VSI	MRVF	VSI	MRVF	VSI	MRVF	VSI
1	33.94	19.66	12.88	7.44	2.7	1.71	2.49	1.49
2	28.68	59.31	8.53	12.92	3.10	1.15	2.45	0.38
3	48.61	14.84	22.78	8.36	3.55	2.07	3.48	1.03
4	51.39	39.05	34.64	25.31	3.89	3.57	3.17	2.08
5	39.73	38.26	17.83	19.85	3.26	0.70	2.42	0.31
6	58.54	24.34	46.58	17.98	2.53	0.95	1.79	0.46
Average	43.48	32.58	23.87	15.31	3.17	1.69	2.63	0.96
SD	11.31	16.36	14.32	6.98	0.51	1.05	0.60	0.71

**Table 4:** Root mean square deviations (RMSDs) calculated for the whole brain and tumor regions between the vessel radius (in μm) and rCBV (in %) maps obtained from the raw and noisy datasets for the MRVF and VSI techniques. The average and SD of the RMSD for each parameter and technique among the 6 subjects are also shown.

Subject	RMSD of Vessel Radius (μm)				RMSD of rCBV (%)			
	Whole Brain		Tumor		Whole Brain		Tumor	
	MRVF	VSI	MRVF	VSI	MRVF	VSI	MRVF	VSI
1	28.92	60.10	18.97	6.01	0.81	1.21	0.31	1.27
2	21.66	136.67	8.62	7.26	0.71	0.79	0.37	0.05
3	34.12	21.15	30.3	7.02	1.02	1.04	1.23	0.29
4	27.32	62.33	23.31	16.19	1.43	1.82	1.56	0.13
5	26.32	63.95	19.84	12.86	0.63	0.25	0.51	0.04
6	29.35	41.31	34.54	41.26	0.71	0.47	0.72	0.06
Average	27.95	64.25	22.59	15.10	0.88	0.93	0.78	0.31
SD	4.09	39.14	9.15	13.41	0.30	0.56	0.51	0.48

## Data Availability

Data are available on request.
